# Processive Pathways to Metastability in Block Copolymer Thin Films

**DOI:** 10.3390/polym15030498

**Published:** 2023-01-18

**Authors:** Nayanathara Hendeniya, Kaitlyn Hillery, Boyce S. Chang

**Affiliations:** Department of Materials Science and Engineering, Iowa State University, Ames, IA 50011, USA

**Keywords:** block copolymer, metastable, solvent annealing, thermal annealing, post-processing, nonequilibrium, self-assembly

## Abstract

Block copolymers (BCPs) self-assemble into intricate nanostructures that enhance a multitude of advanced applications in semiconductor processing, membrane science, nanopatterned coatings, nanocomposites, and battery research. Kinetics and thermodynamics of self-assembly are crucial considerations in controlling the nanostructure of BCP thin films. The equilibrium structure is governed by a molecular architecture and the chemistry of its repeat units. An enormous library of materials has been synthesized and they naturally produce a rich equilibrium phase diagram. Non-equilibrium phases could potentially broaden the structural diversity of BCPs and relax the synthetic burden of creating new molecules. Furthermore, the reliance on synthesis could be complicated by the scalability and the materials compatibility. Non-equilibrium phases in BCPs, however, are less explored, likely due to the challenges in stabilizing the metastable structures. Over the past few decades, a variety of processing techniques were introduced that influence the phase transformation of BCPs to achieve a wide range of morphologies. Nonetheless, there is a knowledge gap on how different processive pathways can induce and control the non-equilibrium phases in BCP thin films. In this review, we focus on different solvent-induced and thermally induced processive pathways, and their potential to control the non-equilibrium phases with regards to their unique aspects and advantages. Furthermore, we elucidate the limitations of these pathways and discuss the potential avenues for future investigations.

## 1. Introduction

Research and development on block copolymers (BCPs) have experienced a massive growth in the past few decades due to (i) their application in various fields, and (ii) the potential platform for fundamental research into the nature of self-assembly. There is no doubt that current BCP architectures are incomparable to biopolymers (i.e., proteins and DNA), however, they provide the necessary building blocks for us to comprehend (and appreciate) the complexity and hierarchical nature of the molecular machines. From inception as surfactants [1] research in BCPs have evolved into more advanced areas, including battery electrolyte membranes [2,3], advanced lithography [2,4,5,6,7], nanocomposites [8,9,10,11], ion-exchange membranes [12,13], nanopore templates [14], and patterned surfaces [15,16].

**Figure 1:**
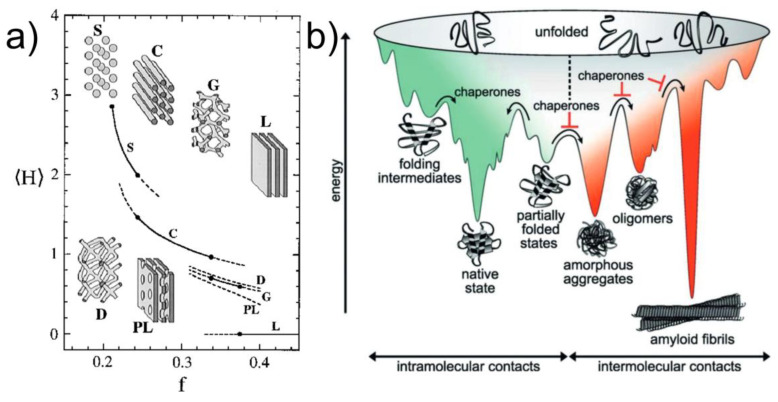
(**a**) Schematic of a diblock copolymer curvature phase diagram. The average mean curvature, <H> dictates the equilibrium phase as it compensates for the entropic loss due to chain stretching between the blocks at asymmetric volume fractions, f << 0.5 [19]. (**b**) Energy landscape of protein folding highlighting the myriad of metastable states accessible [23]. Adapted with permission from references [19,23].

BCPs self-assemble into body-centered cubic spheres, hexagonally packed cylinders, lamellae, and gyroid phases with critical dimensions ~5–50 nm (Figure 1a) [17,18]. These distinct nanostructures are the result of an equilibrium between the minimizing interfacial area of chemically incompatible blocks and maximizing the conformational entropy of polymer coils [19]. Thus, equilibrium structures are ultimately determined by the chemistry of the repeat units, the relative size of each block, and the chain architecture−all of which affects the block incompatibility characterized by χN (product of the Flory–Huggins interaction parameter and degree of polymerization), and entropic penalty due to chain stretching. As a result, the phase segregated chains form a brush layer along the interface [20,21]. It should be unsurprising, however, that the metastable phases, such as the so-called perforated lamellae and double-diamond morphologies, can be obtained with sufficient kinetic barriers in place (Figure 1a).

While there is evidently a massive emphasis on the equilibrium structures obtained from the parameters above, block copolymers have long been predicted to exhibit unique non-equilibrium characteristics. For example, the energy barrier, ΔF for the formation of the ordered lamellar structures from a kinetically trapped state, is unusually low due to the small interfacial tension between the order-disorder phases. This energy scales with *N*^−1/3^*δ*^−2^ where *N* is the size of the chains and *δ* is the degree of undercooling, meaning the barrier to the phase transition decreases as the molecular weight increases [22].

Metastable structures are often observed in biopolymers, such as proteins, which typically possess a diverse energy landscape consisting of different folded states [23,24]. Separated by kinetic barriers, these states provide the gateway to the protein functionality (Figure 1b). Similarly, harnessing the ability to kinetically trap the BCP metastable phases will create a structural diversity that is unrestricted by the molecular characteristics of the polymer. As such, the development of novel processing methods for controlling non-equilibrium phases is critical, and at the same time quintessential to materials science and engineering.

In this review, we explore the different thin film processing approaches that provide control in the metastable regime. The processing pathways for achieving metastable phases require controlled perturbations that overcome the kinetic barrier (Figure 1b) and quench the mechanisms that pin the system within the phase space. This review, however, is not intended to be an exhaustive summary of the block copolymer self-assembly. We divide the methods for the non-equilibrium processing into two general categories (i) solvent-induced and (ii) thermal-induced pathways. Within each process, the uniqueness of the method is discussed alongside the mechanism for the phase control. This is followed by the limitations, potential improvements, and outlook.

## 2. Solvent-Induced Pathways to the Metastable Phases in Block Copolymer Thin Films

### 2.1. Classic Solvent Vapor Annealing

The early studies of block copolymer self-assembly were focused on thermal annealing. During thermal annealing, elevated temperatures are used to mobilize the polymer blocks to facilitate its self-assembly [25,26]. Thermal annealing is generally a viable option to obtain ordered structures of low-χ block copolymers with a low order-disorder transformation (ODT) temperature. However, high-χ block copolymers with an extremely high ODT point and a low thermal degradation point are not suitable for thermal annealing [27,28]. This can be overcome by using a solvent with favorable interaction parameters. The self-assembly of block copolymers in a solution has been extensively studied using both experimental and theoretical approaches [29]. The addition of a solvent into a block copolymer system provides extra degrees of freedom for the self-assembly. The effect of the solvent on the self-assembly can have four major aspects.

1. The solvent reduces the effective T_g_ of the thin block copolymer film by swelling the film. The addition of the solvent molecules enhances the chain mobility [30,31,32];
2. The adsorbed solvent molecules change the effective volume fraction (ϕ_eff_) of the blocks and therefore, the effective interaction parameter (χ_eff_) between the blocks [33,34,35,36];
3. The solvent also mediates the surface energies. This changes the preferential wetting of the free surface by the different blocks [30,33,37];
4. The addition of a solvent can screen out the undesirable block-substrate interactions and avoid the dewetting of the thin film [30,38].

**Figure 2:**
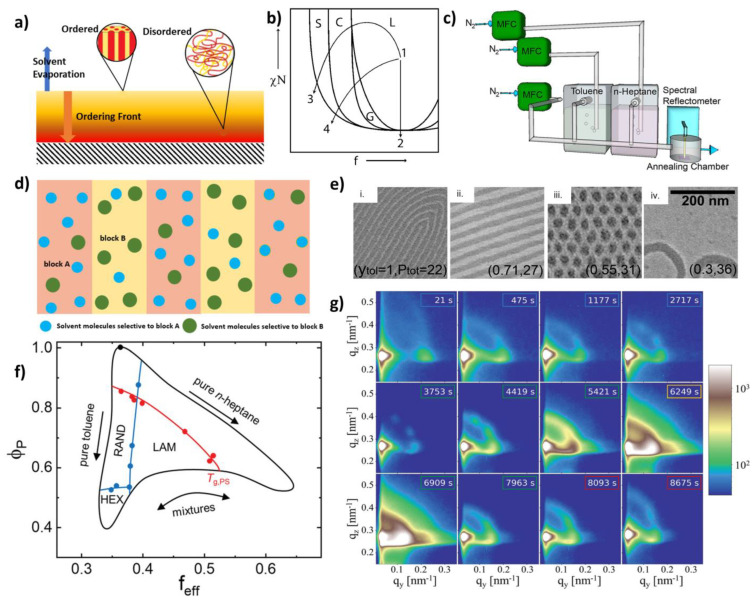
(**a**) Schematic diagram of the solvent evaporation and ordering in a thin block copolymer film, [38]. (**b**) Diblock copolymer phase diagram showing the effect of swelling in different solvents. 1–2: swelling in a nonselective solvent; 1–3, 1–4: swelling in a highly selective and a partially selective solvent [42]. (**c**) Continuous flow annealing with the mixed solvent vapors [43]. (**d**) Schematic representation of the solvent activity inside the thin film, based on the polymer-solvent interaction parameter. (**e**) (i, ii, iii, and iv) Morphologies observed in toluene and n-heptane solvent mixture, depending on the partial pressure and volume fraction of toluene (given in parentheses) [43]. (**f**) Diagram of the states representing different process pathways [44]. (**g**) GISAXS images obtained in the stepwise solvent vapor annealing [44]. Adapted with permission from references [38,44].

These four aspects collectively contribute to deflect the effective domain spacing from the bulk state of the block copolymer, leading to the new domain spacing and interfacial width [39,40]. While this may complicate the self-assembly mechanism, it can also form a desired metastable microphase separated morphology [29]. Navigating through the morphologies is highly dependent on the balancing of the solvent evaporation kinetics against the solvent diffusion kinetics [36]. In the beginning, the rapid solvent evaporation occurs at the thin-film surface, leading to a concentration gradient and subsequently, the ordered morphologies, leaving the disordered structure in the interior of the film. With time, more solvent evaporates from the interior, allowing the film to have an ordering gradient that propagates into the film (Figure 2a) [30]. A broad range of geometries and feature sizes can be obtained from the solvent vapor annealing without the need to change the molecular weight or volume fraction [41].

Use of the solvents in the self-assembly of block copolymer thin films is an effective and highly directional method of navigating the microphase separation by controlling the solvent selectivity and evaporation rate [34,38,45,46,47]. The final morphology and orientation of the thin film is highly dependent on the type of solvent being used [30,45,48,49,50,51,52]. A range of phases can be observed by dissolving a BCP in a low molecular weight selective solvent. The morphologies obtained from the solvent-cast and solvent annealed thin films are not at equilibrium, in general due to the rapid evaporation kinetics. Hence, there has been a major interest in the phase behavior of the block copolymer self-assembly in the selective solvents. The solubilization of the low molecular weight solvent molecules in the diblock copolymer micelles has been developed by Nagarajan and Ganesh [53]. A block-selective solvent is a good solvent for one block and can be neutral or poor for the other block, leading to the formation of micelles [54]. The interaction between the solvent and each polymer block accounts for the effective interaction parameter of the system. Depending on the solvent selectivity (non-selective, partially selective, and highly selective) the block copolymer may take different kinetic pathways through the phase diagram [42] (Figure 2b). The selective solvent swelling allows the dynamic pathways to explore the morphological evolution of the BCPs [55]. Furthermore, the selective solvents allow for the creation of a controlled orientation, a widely explored idea as a promising method for nanolithography i.e., directed self-assembly (DSA) [41].

Control of the feature size is one of the most significant aspects of the selective solvent vapor annealing. Park and colleagues used tetrahydrofuran as the annealing solvent (selective to PS block) on polystyrene-block-poly(4-vinylpyridine) thin films and observed the decreasing microdomain size and domain spacing with a significantly enhanced lateral order of the cylindrical microdomains against the increased annealing time [47]. The changes in the domain spacing was also observed for poly(2-vinylpyridine-block-dimethylsiloxane) (P2VP-b-PDMS), using a range of solvents including polar solvents, which contain alcohol and acid groups that have a high affinity to 2VP block [41]. The morphologies obtained from the solvent annealing remained after quenching the thin films, by exposing them to dry air.

The choice of different solvents affected the degree of swelling. Consequently, the mobility of the BCP chains increases. Different solvents used in this study showed a varying selectivity to the blocks of the polymer. As a result, the swelling induced by each solvent is different. The diffusion of the solvent molecules into a particular block is effectively increasing its volume fraction. Therefore, the BCP thin film can undergo a range of morphological transformations, depending on the annealing time. However, the molecular weight or the composition of the BCP remains intact. This highlights one of the major advantages of solvent annealing. The importance of the rapid solvent evaporation or quenching is also highlighted in this study, as it allows to preserve the metastable structures by kinetic trapping.

The use of mixed solvent vapors to instigate the microphase separation in block copolymers was first demonstrated by Jung and Ross [36]. They achieved the systematic tunability of the microphase-separated morphologies of polystyrene-*block*-polydimethylsiloxane (PS-b-PDMS) thin-films using n-heptane (selective to PDMS block) and toluene vapors in an annealing reservoir. The cylinder-forming PS-b-PDMS can be transformed into perforated lamellar morphology by increasing the n-heptane volume fraction. This was attributed to the reduced effective χ resulting from the preferential swelling of the minority PDMS block and resulting thermodynamic forces that drive the phase separation forward. The study also mentions that lower vapor pressures may not induce enough chain mobility to achieve an equilibrium morphology, which may be an unfavorable outcome regarding nanopatterning. However, it is a promising observation to further discover the non-equilibrium morphologies obtained in lower solvent vapor pressure regimes. Park et al. also studied the effect of the volume ratio of the solvents on the morphological transformation of the BCP in a binary solvent system. They used dimethylformamide (DMF) and toluene vapors on a cylinder-forming poly(styerene-b-2-vinylpyridine) (PS-b-P2VP) block copolymer and obtained line, honeycomb, circular hole, and lamellar morphologies by precisely controlling the volume ratios of the solvents [36]. By changing the volume ratio between DMF and toluene, the volumetric fraction of the BCP can be varied. The increased V_DMF_/V_Toluene_ ratio drives the swelling of the P2VP block in the forward direction. As a result, the effective volume fraction of the P2VP block increases. This leads to morphological changes starting from the circular and goes through the perforated lamellar to the lamellar structure. At V_DMF_/V_Toluene_ = 0.62, they observed the honeycomb structure with a hexagonal hole, due to the uniform line thickness preference of the P2VP block. As the solvent volume ratio decreased, the line width of the honeycomb structure was observed to be decreased, along with the discrete formation of the P2VP network. In a later study, they showed that the increased selectivity of the preferential solvent in the mixture can facilitate the self-assembly with the increased domain spacing, which suggests the increased effective interaction parameter (χ) by using the toluene and heptane solvent mixture on PS-b-PDMS BCP [56]. Consequently, the effective interaction parameter improves and thereby the domain spacing (D), which correlates to χ as D≈ χ^1/6^ [36]. By changing the solvent volume ratio, the ordering of the pattern can be improved. This refers to the ability of a solvent to selectively decrease the activation barrier of chain diffusion.

The classical approach in conducting the solvent vapor annealing is to use solvent reservoirs. In this method, a thin film is placed on a stage inside a closed jar that contains the solvent in liquid form. Highly volatile solvents immediately form a vapor phase, and the solvent molecules start to diffuse into the thin film. However, the use of solvent annealing reservoirs imposes several limitations in controlling the crucial process parameters, such as the partial vapor pressure of the solvents in the mixture. The annealing reservoirs restrain the independent control of the partial pressure of each solvent. The partial pressures of each solvent in the vapor phase cannot be easily quantified. Furthermore, the solvent activity in the vapor phase is transient, limiting the control over the solvent activity inside the thin film. The continuous flow vapor annealing systems overcome these challenges. By using a continuous flow system, one can attain a simultaneous flow of vapors and enhanced mixing. Furthermore, the increased flow control results in the increased control of partial pressures of the solvent vapors. Figure 2c schematically represents a flow-controlled solvent vapor annealing system. The mass flow controllers utilized in this assembly allow the user to systematically control the partial vapor pressure of each solvent, leading to a rich variety of mixed solvent compositions and subsequently, a more elaborate phase behavior. The affinity of the solvent molecules to a block is defined by the polymer-solvent interaction parameter. This parameter is derived from a relationship between the solubility parameters of the solvent and the polymer. Solvents that have similar solubility parameters with the polymer, tend to diffuse into their preferred polymer block. This leads to the preferential swelling of the block. However, due to the mixing entropy, the less-favorable solvent partially diffuses into the polymer block in small amounts, as depicted in Figure 2d. Thus, the preferential swelling is a convenient way of manipulating the swelling of the blocks, and consequently, the resulting morphologies.

Gotrik and colleagues used a continuous flow system to study the effect of a mixture of solvent vapors on the morphological transformations of polystyrene-*block*-polydimethylsiloxane (PS-b-PDMS) thin films, by varying the flow rates of the toluene and n-heptane vapors [43]. A phase diagram was constructed with reference to the partial pressure of toluene against the total vapor pressure inside the annealing chamber. The results were then compared with traditional reservoir solvent vapor annealed PS-b-PDMS thin films in toluene and n-heptane vapors. The study signified the use of continuous flow systems by accessing regimes of solvent vapor pressures otherwise inaccessible, to obtain a range of morphologies, as depicted in Figure 2e, by carefully controlling the partial vapor pressures of the two solvents [43]. Non-ideal solvent mixtures can be described by using the non-random two-liquid model (NRTL) which correlates the activity coefficient of a solvent (*γ*) with the molar fraction in the liquid phase (Equation (1)) where 1 and 2 refer to the two solvents, *x* is the molar fraction in the liquid, and *α* and τ are the experimentally determined coefficients.

(1) $$ln\gamma_{1}=x_{2}^{2}\left[\frac{\tau_{12}e^{-\alpha_{12}\tau_{12}}}{{\left(x_{2}+x_{1}e^{-\alpha_{12}\tau_{12}}\right)}^{2}}+\frac{\tau_{21}e^{-2\alpha_{12}\tau_{12}}}{{\left(x_{1}+x_{2}e^{-\alpha_{12}\tau_{21}}\right)}^{2}}\right]$$

Compared to a solvent reservoir, the continuous flow systems can maintain a range of partial pressures and allow equilibration. This allowed a wide range of morphologies, otherwise inaccessible. Furthermore, the lower partial vapor pressures are crucial in annealing the low molecular weight BCPs.

Chavis et al. also used a continuous flow system with mixed solvent vapors of methanol and tetrahydrofuran on poly (2-hydroxyethyl methacrylate)-*block*-poly (methyl methacrylate) (PHEMA-b-PMMA) block copolymer thin films. The lamellar morphology was observed in the bulk form, and transitioned into ordered lamellae, gyroid, hexagonal, and spherical morphologies in thin film. The study attributes the results to the increased mobility of the individual polymer chains provided by the solvent molecules. The authors also highlight the importance of the polymer-solvent interaction parameter, which governs the affinity of a certain block with a solvent. This ultimately defines the number of solvent molecules that diffuse into a block. It is also important to have similar vapor pressures, melting points and boiling points, for the convenience of handling the solvent fractions in the mixture. The in-situ characterization methods (gracing incident small angle X-ray scattering (GISAXS) and reflectometry) are used to study the transition of the block copolymer films from one initial disordered morphology to different ordered morphologies [57,58]. The rapid solvent evaporation can kinetically trap the metastable structures [45,59]. Chavis et al. also highlight the changes in the domain spacing due to the film swelling and deswelling. They observed the increased domain spacing (32 nm) during the swelling and the decrease in the domain spacing (15 nm) to more than half of the swelled domain spacing after the solvent molecules are completely evaporated. This is lower than the pre-annealed lamellar domain of the film (29 nm), which suggests that the observed lamellar structure after SVA is in fact, metastable.

Furthermore, Berezkin and colleagues used a versatile solvent annealing set up that allows the user to vary the sample temperature, in addition to the vapor pressure and composition of the vapor mixture. By utilizing the in-situ GISAXS characterization of the swollen film which further facilitates the immediate and efficient identification of the SVA parameters [60]. Further, they utilized molecular simulations to calculate 2D the GISAXS maps for the BCP films with certain morphologies, as an approach of verifying their experimental results. The ease of navigating through the phase diagram offered by this method is a more promising pathway to achieve the metastable phases (Figure 2f). Following the work of Shelton and colleagues [61], this study also emphasizes the use of 2D GISAXS (Figure 2g) maps to extract information about the solvent distribution in the BCP nanodomains. This approach provides a window to discover the actual solvent distribution in each domain, which is a major challenge in using mixed solvent vapor annealing. Jung et al. highlighted the importance of the process pathway to achieve the desired morphologies, following the work of Berezkin [44]. In this study, three pathways have been explored, one of them being the solvent exchange. First, the thin film is swollen in one solvent and then it is exchanged by the other solvent until a desired ratio is reached. The exchange of solvents allows each block to swell in its most preferred solvent. This approach is important in controlling morphologies. The exchange of solvents is a novel approach with several different possibilities. In addition to what is presented in this study, a swelling solvent can be exchanged with a non-swelling solvent. By doing so, the thin film can be collapsed, hence, quenched. This has the potential to be used as a method of preserving the metastable structures. It should be noted that the solvent exchange rate is the most critical parameter as it determines how rapidly the film deswells. The effect of the solvent exchange rate on preserving the morphologies and the further morphological evolutions is a realm that needs further understanding.

### 2.2. Direct Immersion Annealing

The conventional solvent vapor annealing is a promising pathway to obtain the long-range order in the high-χ block copolymer thin films, along with the added advantage of the easy navigation through various metastable morphologies. However, the solvent vapor annealing chambers impose several challenges, especially when considering the scale-up of this process at the industrial level for nanofabrication. The swelling behavior of the thin films in a solvent annealing chamber is highly dependent on the nature of the solvent and the extent of the solvent adsorption. Film swelling is directly governed by the solvent vapor activity inside the film. The direct and accurate measurement of this parameter is a cumbersome task. Therefore, it is challenging to correlate the solvent activity inside the film and the film swelling, which may affect the reproducibility. Film swelling can also involve some undesirable effects, such as dewetting, differential block swelling, and shifts in the order-disorder and/or order-order transformation [62,63,64]. The preservation of the solvent annealed morphologies is achieved by quenching. Depending on the processing conditions, the time scale of the quenching may vary from minutes to several days [65]. These complexities call for the need for an annealing technique that is amenable to be scaled up with fewer variables to control.

**Figure 3:**
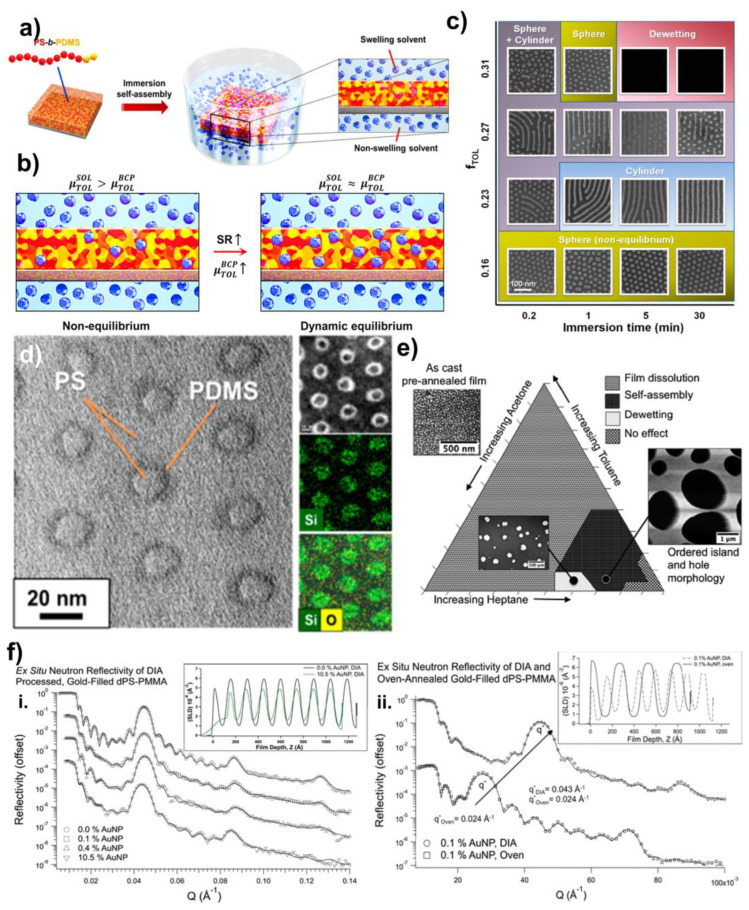
(**a**) Concept of the immersion-induced solvent annealing/direct immersion annealing (DIA) [69]. (**b**) Illustrations of the different swelling states and the comparison of the chemical potential inside the film with the increasing swelling ratio [69]. (**c**) Self-assembled morphologies of the PS-b-PDMS BCP treated with different solvent compositions and annealing times [69]. (**d**) Formation of the core-shell structures during the two-step annealing [69]. (**e**) Ternary phase diagram representing the solvent ratio of the solvent mixture used in direct immersion annealing, based on its effect on the BCP thin film [70]. (**f**) (**i**,**ii**) Neutron reflectivity data representing the reduced domain size of the PS-b-PMMA BCP thin films during direct immersion annealing [70]. Adapted with permission from references [69,70].

The use of the immersion induced controlled dewetting of thin homopolymer films, was first demonstrated by Sharma and colleagues [66,67,68]. An extension of the immersion annealing to the block copolymer thin films was first investigated by Park et al. [69]. The immersion-induced self-assembly or direct immersion annealing (DIA) is achieved by immersing a spun-cast block copolymer thin film in a mixture of solvents (Figure 3a). The thin film is the reactive ion etched following the annealing to reveal the self-assembled morphology [65,69,70]. Compared to the complex flow controlled solvent annealers, direct immersion annealing simplifies the self-assembly of the BCP thin films to a greater degree. The use of a solvent mixture in this process serves several purposes. The solvent mixture consists of a non-swelling and swelling solvent. The choice of solvent depends on the hydrophilicity of the blocks and the solubility parameters. A poor solvent is chosen as the non-swelling solvent, to regulate the dissolution of the polymer thin-film [65]. Usually for the hydrophobic blocks, the non-swelling solvent can be a polar solvent, such as ethanol or methanol [69]. However, Modi and colleagues reported that the small molecule solvents, such as alcohols, may not be suitable for the BCP systems, due to their high mobility and the resultant substrate induced delamination of the film [65,71]. The good solvent in the mixture imparts the mobility of the block copolymer chains and induces swelling. Depending on the hydrophilicity and the solubility of the block, the appropriate solvent is chosen [69]. The miscibility of the selected solvents at a given temperature, is highly important in obtaining a homogeneous mixture [65].

The swelling ratio of the thin film is dependent on the chemical potential inside the film. At the beginning of the immersion annealing, the chemical potential of the solvent mixture is greater than the chemical potential of the solvent inside the BCP, and therefore, the system is not in equilibrium. As the solvent molecules plasticize the polymer, the swelling of the film increases and eventually, the chemical potential of the solvent inside the BCP and solvent mixture come to a dynamic equilibrium point. The increased swelling solvent fraction increases the tendency in the equilibrium swelling ratio (Figure 3b). The careful selection of the solvent mixing ratio provides the ability to control the swelling of the film, and consequently, the morphologies [69]. The morphologies obtained in direct immersion annealing are highly dependent on the immersion temperature and immersion time, in addition to the solvent mixing ratio (Figure 3c). The DIA can result in rapid and stable morphologies, and they are closely in accord with the predicted equilibrium morphologies. Furthermore, this technique can be applicable to the loosely segregated BCP systems (low χ), such as polystyrene-b-poly (methyl methacrylate) (PS-b-PMMA), and the highly segregated BCP systems, such as polystyrene-b-poly (2-vinylpyridine) (PS-b-P2VP) [65].

Park and colleagues demonstrated that the immersion annealed PS-b-PDMS thin films in the heptane/ethanol mixture (selective to PDMS block) resulting in the core-shell sphere and the core-shell cylinder structures, that are different from the morphologies obtained from immersing the same BCP in toluene/ethanol (selective to the PS block) (Figure 3d) [69]. These structures were not observed when the same film was annealed in a classic solvent vapor annealing chamber. The higher degree variety of the morphologies in DIA was attributed to the increased volume fraction of the PDMS block and the high selectivity of the solvent mixture at the polymer–solvent interface. Therefore, DIA is a good way to obtain a greater tunability of the BCP morphologies. Furthermore, a two-step DIA can induce the improved order in these core-shell structures. First, the film is swelled in the toluene/ethanol mixture to obtain the spherical dots, and these can be converted into well-organized core-shell spheres, followed by swelling in a heptane/ethanol mixture. The core-shell structures are indeed metastable, as they progress to convert into a perforated lamellar structure, as the annealing time is increased. Annealing using heptane with a solvent fraction, f_HEP_ = 0.056 for 10 s, forms a continuous PDMS layer filled with perforations of PS chains. As the treatment time increased, the PS perforations became more regular and achieved a long-range order. Furthermore, the core-shell spheres can be reinstated to their original spherical morphology by annealing in a toluene/ethanol mixture.

Modi and colleagues explored the significance of the solvent selection for direct immersion annealing using PS-b-PMMA as the BCP and acetone/n-heptane as the solvent mixture (Figure 3e). Selecting a solvent mixture for a low χ BCP is a complicated task. Near equally good solvents for both blocks can significantly reduce the effective χ between the blocks. Lowering the χ to weaken the segregation limit may be useful, as it can accelerate the kinetics of the defect removal, leading to the long-range order [65]. DIA results in well-ordered cylindrical patterns within seconds, at temperatures well below the glass transition of both blocks of the BCP. Compared to that, conventional thermal annealing takes hours to give a similar ordering at modest temperatures above T_g_. The activation energy for the grain growth in direct immersion annealing is lower than the activation energy for the defect diffusion reported in early studies [72], suggesting that DIA is a convenient way for the morphological transformations. The composition selection for the solvent mixture is governed by a complex interplay of the molecular weight of the BCP, block composition, annealing temperature, film thickness, and substrate energy. Although the experimental swelling ratio follows a linear relationship with the acetone fraction, the theoretical swelling ratio for this system significantly deviates from the experimental data. The authors attribute this scenario to the high selectivity of acetone towards the PMMA block and the resulting deviation of the dependance of χ on the solvent quality.

Another important aspect of direct immersion annealing is obtaining the reduced domain size and the interfacial width [70]. Longanecker et al. examined this scenario by comparing the total domain spacing (L_0_) of direct immersion annealed PS-b-PMMA BCP thin films with the conventional thermal annealed thin films of the same (Figure 3f). They observed that the domain spacing of the immersion annealed thin films are significantly lower (14.7 nm) than the thermally annealed thin films (25.9 nm) tracked using neutron reflectivity. The thin films have a restricted expansion in the lateral direction during swelling. In the disordered state, approximately half of the chains assemble next to each other to accommodate the larger volume of the swollen chains. Thus, the remnant chains compensate by forming more layers on top, compared to the thermally annealed films. When the solvent is removed rapidly from the thin film, these layers collapse back down without significantly changing the lateral junction density. This leads to thinner layers and thereby a reduced domain spacing. This reduction in domain spacing is crucial, as it serves as a pathway to create smaller feature sizes. The reduced domain spacing suggests a metastability of the resulting structures.

### 2.3. Solvent Drying

One of the major potential applications of block copolymers is in additive manufacturing. However, in general, the additive manufacturing of functional materials possesses complications, due to the limited control of the microstructure and the assembly at the nanoscale. Direct immersion annealing, as discussed in the previous section, is a promising realm for further discoveries, as a way of utilizing block copolymers in additive manufacturing. However, DIA still possesses limitations, due to its very nature of incorporating solvent mixtures. The annealing solution should be carefully catered for or otherwise, the tunability of the resulting structures may differ from expectations. Furthermore, careful attention should be paid to how to prevent the delamination of the film. In addition to that, the complete evaporation of the solvent after annealing, is a crucial post-processing step to preserve the morphology. This involves purging an inert gas onto the film or drying in a vacuum oven well-below the T_g_ of the BCP. Therefore, a substantial interest in a simpler technique is called for, regarding the additive manufacturing of BCPs.

**Figure 4:**
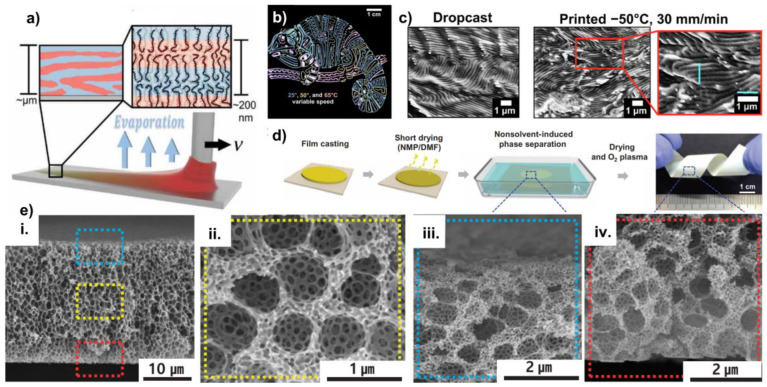
(**a**) Schematic diagram of the concept of the direct-write 3D printing [74]. (**b**) Change in photonic properties of the BBCP photonic crystals, depending on the temperature and printing speed [74]. (**c**) Formation of the morphologies during the drop casting and 3D printing [74]. (**d**) Concept of solvent drying for the formation of hyperporous nano membranes [75]. (**e**) (**i**–**iv**) Membrane morphologies along the lateral direction of the film [75]. Adapted with permission from references [74,75].

3D printing technologies are commercialized widely and are used for the melt extrusion of thermoplastic structural polymers, due to the simplicity of the method, low cost, and high reliability [73]. Patel and colleagues comprehensively studied the self-assembly kinetics of the bottlebrush block copolymers, by precisely directing the materials during the non-equilibrium 3D printing. This technique compensates for most limitations of direct immersion annealing and most importantly, introduces alternative additive manufacturing techniques that can be used for BCPs. Three-dimensional printing integrates both hardware and software, which allows the user to manipulate the composition of the BCP solution carefully and precisely. The ease of manipulation can be utilized to obtain a range of metastable phases, as depicted in the work by Patel and colleagues, with regards to the bottlebrush block copolymer (BBCP) photonic crystals (PCs) with color changing ability [74]. Furthermore, it only requires a dissolving solvent to continue the process, unlike traditional solvent vapor annealing and direct immersion annealing, where a swelling solvent or a mixture of solvents are required to induce the chain mobility. Film casting and self-assembly are both completed in one step (Figure 4a). Although depositing thin films from a solution adds an additional degree of freedom to the phase diagram, it is not overly complicated and sufficiently imparts the molecular mobility at lower temperatures and can be used to lever the highly accelerated self-assembly. Furthermore, the use of software creates a flexible interface between the user and the hardware and gives the user more freedom to adjust the parameters.

Direct-write 3D printing from a solution, is not only convenient, but permits the ability to manipulate the self-assembly of the BCP to a greater degree. Three dimensional printed PCs of BBCPs have well-controlled, unform, and highly tunable photonic properties. Easy control of the process parameters, such as the printing speed, applied pressure, and substrate temperature, can be used to control the photonic properties (color) of the films. It can also be used to create the complex patterns that possess a higher degree of spatial and functional control with the changing color, as depicted in Figure 4b. The authors attribute the variety of photonic properties to the domain size control of the underlying lamellar morphology of the BCP thin films. They observed that the films deposited under different temperatures and printing speeds, possessed a lamellar morphology with varying correlation lengths and orientations. Direct-write 3D printing allows the lamellae grains to orient uniformly and hence the uniformity of color at a given temperature and printing speed. To test this hypothesis, they have also analyzed the microstructure of a drop casted film, which revealed a dispersed orientation that led to the color dispersity of the film (Figure 4c). However, the authors did not quantify the domain spacing of the 3D printed films, which may have revealed more information in relation to the photonic properties. Printing at higher speeds leads to a blueshift in color that is proposed, as due to the trapping of the metastable, smaller lamellar domain spacings. By allowing a series of printed (under different printing speeds) thin films to equilibrate under classic solvent vapor annealing before the removal of solvent, this kinetic hypothesis is confirmed. Solvent vapor annealing eliminates the effect of the differential solvent removal and causes the domain spacing to converge. This study opens new pathways of combining self-assembly and processing, which is a crucial measure in utilizing the functional materials in the industry level.

Solvent drying is not only applicable for 3D printing. Yoo and colleagues have showed that the hierarchical multiscale hyperporous membranes (HMH-BCP membranes) can be formed in polystyrene-block-poly (4–vinylpyridine) (PS-b-P4VP) thin films, by introducing a surface energy modifying agent into the pore forming block (in this case P4VP) through the nucleophilic substitution, followed by controlling the short drying step, non-solvent polarity, and solvent drying time [75]. This is a nonconventional approach to the solvent annealing of BCPs, that combines the aspects of attaching small molecules to a polymer block, the affinity of the different blocks to the solvent being used, and the solvent drying (Figure 4d). The porous structures are indeed thermodynamically metastable, due to their large surface area. In this study, the authors demonstrate the ability to obtain the porous structures with both macro- and nano-phase separation. Although the focus of this study is to demonstrate the effect of the nucleophilic substitution of the small molecule on macro- and nano-phase separation, a considerable attention was paid to the type of nonsolvent used in the coagulation bath and short drying time after the film casting, which are more relatable for the focus of this review. They observed that when water is used as a nonsolvent, instead of ethanol, poorly developed smaller nanopores were developed (Figure 4e). This was attributed to the higher polarity of water, which makes it less compatible with the minority block. This mismatch in miscibility leads to poorly developed nanopores. Shorter drying times allow the BCP membrane to contain larger amounts of solvent, leading to the formation of the macro pores by the solvent-non solvent exchange in the coagulation bath. In contrast, with a longer drying time, more solvent is evaporated from the membrane, leading to the low possibility of the solvent-non-solvent exchange, which leads to a film with little to no macropores. This proves that the solvent drying kinetics plays a crucial role in forming the hyperporous structures.

### 2.4. Limitations and Mitigations

To summarize the previous three sections, we discussed the solvent-induced pathways to achieve the metastable phases in the block copolymer thin-films, both in conventional and non-conventional approaches. Although each technique can mitigate the limitations of another technique, to some extent, the solvent-induced pathways to the metastability come with the common inherent limitations, owing to the use of solvents. The organic solvents used in the solvent-induced phase separation are highly volatile. These solvents can be hazardous, if not for the special processing conditions and safety measures used in processing. In the laboratory scale, this can be achieved by using fume hoods and exhausts, followed by personal protective gear for the users. However, the scaling-up of these techniques is limited by, not just the complexity of the processing, but also by the potential health hazards imposed by the process ingredients. Volatile solvents call for specially designed processing facilities, which may not be economically favorable. Furthermore, annealing assemblies, especially in the case of traditional solvent vapor annealing, must be carefully maintained to prevent contamination by the solvent vapors.

Another limitation is presented by the limited options for the choice of solvent. The choice of solvent is governed by the polymer–solvent interaction parameter which relates to the solubility parameters of both the solvent and the polymer, as discussed previously. Several studies highlight the importance of carefully selecting the solvents to govern the phase separation of the BCP thin film, in relation to the solubility parameters [36,43,58,65,69]. However, the organic solvents that are commonly used in the solvent-induced phase separation, are only compatible with the polymer, to some extent. Fully compatible solvents may present the ability to govern the phase separation and formation of the metastable structures more precisely. One alternative for the enhanced compatibility of solvents, is to use a liquid form of the monomer of a certain block, as the preferential solvent. Park and colleagues explored this idea in one of their studies. Due to the high affinity of pyridine to the P2VP block in polystyrene-block-poly (2–vinylpyridine) they observed that small quantities of pyridine can significantly affect the self-assembly kinetics of the BCP with smaller molecular weights, by lowering the activation energy for the chain diffusion [45,76]. This observation provides new avenues of using monomers as selective solvents.

To reveal the morphologies obtained from the solvent-induced self-assembly, the complete evaporation is mandatory. The removal of the solvent is a crucial measure of preserving the metastable structures. The solvent removal is achieved by purging an inert gas on the self-assembled thin film or pulling a high vacuum from the annealing chamber. Inducing a higher degree of chain mobility in the BCP thin films requires a considerable amount of solvent vapor molecules. Quenching may not completely remove the solvent molecules. To mitigate this effect and to control the amount of solvent used, the introduction of non-volatile small molecules to the block copolymer can be an alternative. These molecules can plasticize the block copolymer and induce some degree of chain mobility in the solution. However, this may affect the initial morphology of the cast thin film, and the amount of solvent needed to obtain a desired morphology may differ. This occurs due to the changes in the overall chemical affinity of the film to the solvent molecules.

## 3. Thermally Induced Pathways to Achieve the Metastable Phases in the Self-Assembling Block Copolymers

### 3.1. Microwave Annealing

**Figure 5:**
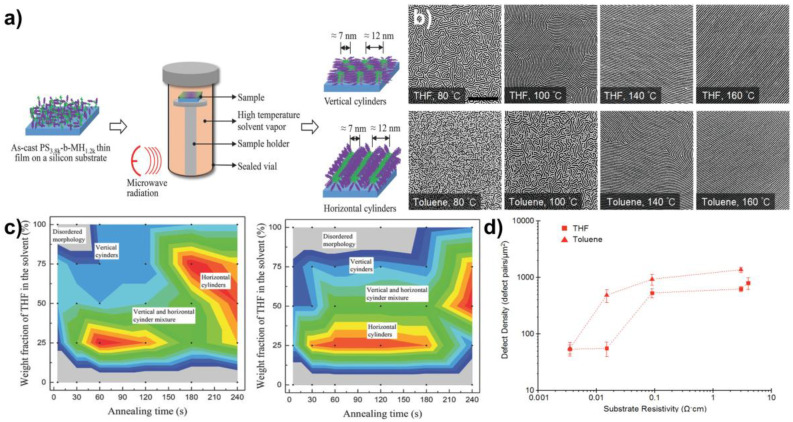
(**a**) Schematic representation of the microwave assisted solvent annealing and reorganization of the BCP morphologies [84]. (**b**) The effect of the annealing solvent on the formation of the well-ordered morphologies in the PS-b-PMMA films during the microwave-assisted solvent annealing [77]. (**c**) Evolution of the morphology with time, under different temperatures (left: 130 °C, right 160 °C), during the microwave-assisted solvent annealing (gray: disordered; blue: vertical cylinders with vertical orientation; green: orientation transition area; red: horizontal cylinders) [84]. (**d**) Plots of the evolution of the defect density vs. the substrate resistivity. Dotted lines not intended to convey any interpolated information [77]. Adapted with permission from references [77,84].

Traditional thermal annealing/oven annealing poses a few limitations on self-assembly, considering the extended annealing times and the difficulty of choosing annealing temperatures for high χ block copolymers [77,78]. As the χ increases, the diffusion coefficient will be decreased, which results in longer times to assemble into ordered structures. Furthermore, high χ BCPs have higher order-disorder transition temperatures (ODTs) which may exceed their degradation temperatures. This imposes another barrier in using oven annealing to induce the self-assembly in high χ BCPs. In contrast, solvent vapor annealing may present a promising pathway to achieve self-assembly under lower temperatures, but still requires longer annealing times to achieve the long-range order [78]. Microwave annealing has garnered more attention in that regard, due to its inherent simplicity. It is a non-traditional and a rapid heating method which is ideal for the thermal and solvothermal annealing of block copolymers [79,80,81,82,83]. Microwave heating can be utilized either to heat the Si substrate that the BCP thin film is deposited on or to heat the solvent reservoir to create a high temperature solvent vapor environment [84,85]. The final morphology is highly dependent on the heating time and the position of the sample within the microwave field. Microwaves are absorbed either by the thin film substrate (wherein most cases, Si wafers with sufficient doping and resistivity) [85] or the polar solvent molecules [86]. The temporal thermal history of the polymer films is changed by the microwave irradiation, leading to the morphological rearrangements. The BCP thin film is placed inside a reaction vessel and, depending on the requirement, the reaction vessel may or may not contain a solvent. Subsequently, the reaction vessel is placed under microwave irradiation, which passively induces the BCP chain mobility and the mobility of the solvent molecules (when a solvent is present) [77,84] (Figure 5a).

Early work on the microwave annealing of block copolymer thin films, was presented by Zhang and colleagues. They have used a commercial microwave reactor to anneal polystyrene–block–polymethylmethacrylate (PS-b-PMMA) and polystyrene–block–poly(2–vinylpyridine) (PS-b-P2VP) BCP thin films to investigate the effect of the substrate resistivity, solvent environment, and annealing temperature on the self-assembly speed and defect density [77]. In this study, they highlighted that the microwave power required for the long-range order depends on the solvent being used. PS-b-PMMA thin films annealed in tetrahydrofuran (THF) at 60 °C, appeared to have rudimentary fingerprint patterns having a domain size expanding from 100 nm to several micron after 60 s, while the same thin films annealed in toluene needed elevated temperatures (140 °C) to achieve a similar degree of ordering, as depicted in Figure 5b. This observation can be attributed to the solvent–polymer interaction parameter. In the case of THF, the PMMA block is more miscible with the solvent, allowing the polymer chains to be plasticized to a higher degree. This allows the PMMA pattern formation, ultimately leading to the highly ordered structures, whereas toluene, being a non-selective solvent for the PMMA block, plasticizes the polymer chains lower than THF. Therefore, higher thermal energies are needed to reduce the energy barrier for the long-range order. High molecular weight polymers require longer ordering times. This suggests that the microwave annealing can navigate high molecular weight BCPs through the phase diagram, more efficiently, and allow for a wide range of metastable phases.

Liao et al. presented an indirect microwave heating technique following the solvent annealing of polystyrene-block-maltoheptaose (PS-b-MH). In their study, neither the thin film nor the substrate is directly irradiated, and the microwave energy was used to irradiate the solvent. The film was mounted on a stage higher above, inside the chamber. However, higher rates of assembly and a similar annealing control was reported, compared to the conventional SVA [84]. The study highlights the convenience of the microwave irradiation in obtaining different morphologies, regardless of the solvent fraction or the selectivity of the solvent. In THF-rich environment (THF/water mixture) BCP thin films resulted in perpendicular cylinders with and without microwave irradiation. However, they noted that the BCP self-assembly was less dependent on the THF weight fraction under the microwave irradiation. This suggests that increased temperatures can excite the morphological evolution where thermally excited solvent vapor increases the chain mobility in the PS-b-MH BCP. By increasing the temperature using microwave irradiation, the phase transition time of PS-b-MH can be reduced from 30 s to 5 s. They also report one of the smallest domain spacing for cylindrical patterns reported so far (12 nm dia.), when annealed using microwave irradiation and a solvent mixture of THF/water = 3/1 (*w*/*w*), suggesting that microwave irradiation technique is a promising pathway to obtain smaller feature sizes. Furthermore, the annealing temperature can modulate the orientation of the morphologies and the rate of phase transitions, as depicted in Figure 5c. Higher temperatures appear to have a greater effect in driving the morphologies in a wide range. This may occur due to the increased chain mobility and rapid structuring, rendering the BCP kinetically trapped in metastable states. However, the THF weight fraction in the annealing solution also affects the morphologies obtained. The THF rich solvent vapor environment leans toward the vertical cylinder morphology, whereas the water rich solvent vapor environment results in the horizontal cylindrical morphology. Although the study does not suggest how the morphological transformation occurs in the BCP, it suggests that the elevated temperature caused by the microwaves is a factor that can excite the orientation transition of the BCP thin films.

Microwave annealing was successfully employed to induce the morphological transformations in a low molecular weight, cylinder-forming PS-b-PDMS (Mn = 16,000 g/mol) BCP [80]. In this study, the researchers used a microwave synthesizer to passively elevate the temperature of a solvent reservoir containing the BCP thin film [80]. The researchers focused on the effect of the annealing time and annealing temperature. Interestingly, the increased annealing time (from 30 s to 180 s) results in more disordered structures with a higher defect density at 323 K. The spun-cast thin films are in a disordered and non-separated state that later orders into regular patterns, induced by microwave energy. Once the thin films reach the upper limit of the ordered regime, they subsequently fall back to a less-ordered state. This is a characteristic of the low Mn block copolymers, where lower χN leads to a weak segregation and therefore the BCP is more likely to have diffuse phase boundaries. This is governed either by the film-substrate thermal gradient or the kinetics of plasticization by the solvent molecules. To test this hypothesis, they studied the effect of increasing the temperatures at a constant annealing time. Although the correlation length of the PDMS cylinders appears to decrease, while the defect density increases beyond the ordered regime with the increased temperature (range 323–423 K), the reduction in the correlation length is not as dramatic as expected. Therefore, it was argued that the microwave irradiation only acts as a passive accelerator for the solvent vapor activity inside the annealing reservoir. The evolution of the morphologies under different temperatures are therefore attributed to the increased swelling of the BCP thin films and the subsequent reduction in the Flory–Huggins interaction parameter, due to the increased solvent vapor activity assisted by the microwave heating.

In another study presented by the same researchers, they have used microwave irradiation without a solvent to induce the pattern formation in the PS-b-PMMA and PS-b-PDMS BCP thin films [81]. Microwave irradiation of spin coated films with sufficient power (~100 W), produced ordered structures, regardless of the molecular weight of the BCPs being used. However, low molecular weight BCP thin films appeared to have the optimum morphological resolution, given their higher diffusion constants, hence, a lower energy barrier during annealing. The critical dimensions of the morphologies obtained in this study are in accord with thermally annealed BCP thin films by the same researchers, further confirming that solvent interactions are crucial for kinetically altering the periodicity of the BCP. In contrast to the previous group, they suggest that microwave energy is responsible for directly inducing the chain mobility rather than passively inducing mobility by elevating the temperature. Therefore, it was concluded that the effective temperature of the film should be much higher to cause thermal annealing, than the measured temperatures of the annealing reactor. However, microwave annealing is more efficient in the pattern formation and morphological evolution when assisted by the solvent vapor annealing and in accordance with the previous observations of Zhang and colleagues [77].

The mechanism of self-assembly under microwave annealing is explored in different studies. Two major mechanisms are proposed for direct microwave annealing and solvent-induced microwave annealing. The mechanism for direct microwave annealing suggests that the substrate resistivity (Figure 5d) is a crucial parameter for the ordered morphologies [85]. Si substrates absorb microwaves, which excite the polymer chains, increasing their mobility. The authors suggest that high resistivity Si substrates absorb more rapidly than the low resistivity substrates. Therefore, higher resistivity substrates are more prone to induce the rapid ordering of structures, hence, the kinetically trapped phases. This approach further suggests that the effect of microwave energy is rather non-thermal, and it is possible to have changes in local electromagnetic fields, due to microwaves causing the self-assembly of the PS-b-PDMS thin films. Mokarian-Tabari and colleagues suggest that the major contribution is made by the rapid interaction of the solvent molecules with microwave energy and the subsequent plasticization of the polymer chains in the presence of solvents [86].

### 3.2. Localized Annealing Methods

The employment of lasers as a directing method for material processing is developing into a higher precision tool for various fields of study [87]. Moreover, lasers are being adapted for the post-preparation reordering of polymeric materials for an array of applications [88]. Some examples include direct laser heating in resins [89], direct laser writing of photonic crystals [90], millisecond ordering of BPC [91] and, laser-induced specialized graphene from polymers [92]. The transient laser heating of polymeric structures is one of many alternative approaches for the nonequilibrium post preparation processing methods of block copolymers (BCPs). Various industries utilize specific and well-defined adaptations of transient laser heating, to adjust and control the hierarchical functionality of the polymeric systems. Methods of post-preparation nonequilibrium laser processing allow for the high tunability of particular features, such as nanoscale morphologies, spatial control heating, and ultrafast phase transformations [87]. Noting the apparent potential of the nanostructures produced by the self-assembly of the BCPs, technical challenges continue to limit the high precision control needed for the bulk uses of thin films within commercial applications. Critical challenges include controlling the nanodomain and morphology, thermal annealing time for self-assembling, and costs associated with the sophisticated instrumentation employed to improve the features [91]. Recently, within the field of soft materials, cold zone annealing (CZA) has been adapted to accommodate the reordering of the morphology in BCPs as a nonequilibrium post-preparation process [87,93]. This unique and newly established method of ordering metastable phases in BCPs, has yielded promising results in recent studies, and may streamline the process of ordering BCP films for industry applications. The defects that persist within the nanodomain after many different forms of thermal annealing, such as LZA annealing methods, zone casting, oven annealing, and microwave irradiation methods, may be relieved by using CZA.

#### 3.2.1. Laser Heating Methods

Successful demonstrations of laser heating for aligning microstructures in block copolymers include laser zone annealing (LZA) and ‘soft-shear’ laser zone annealing (SS-LZA) [91,94]. In a first example, Majewski and Yager demonstrate the advantages of using a laser beam as a heat source on polystyrene-b-poly(methyl methacrylate), PS-b-PMMA, allowing for the highly localized reordering via optical masking. Additional tuning of the spatial and temporal light input field allows for the control of thermal gradient patterns [91]. There are multiple facets to utilize lasers for directed self-assembly of block copolymers that can be employed. Millisecond thermal annealing or laser spike annealing employs a continuous wave (CW) laser focused line that glides over the thin film, rapidly increasing the local temperature and kinetically trapping the sample as it cools. These annealing processes can be tuned by changing the power density, velocity, and shape of the laser, despite the generally expected poor thermal conductivity in the polymers, which results in a consistent long-range order [87,91,94,95,96].

**Figure 6:**
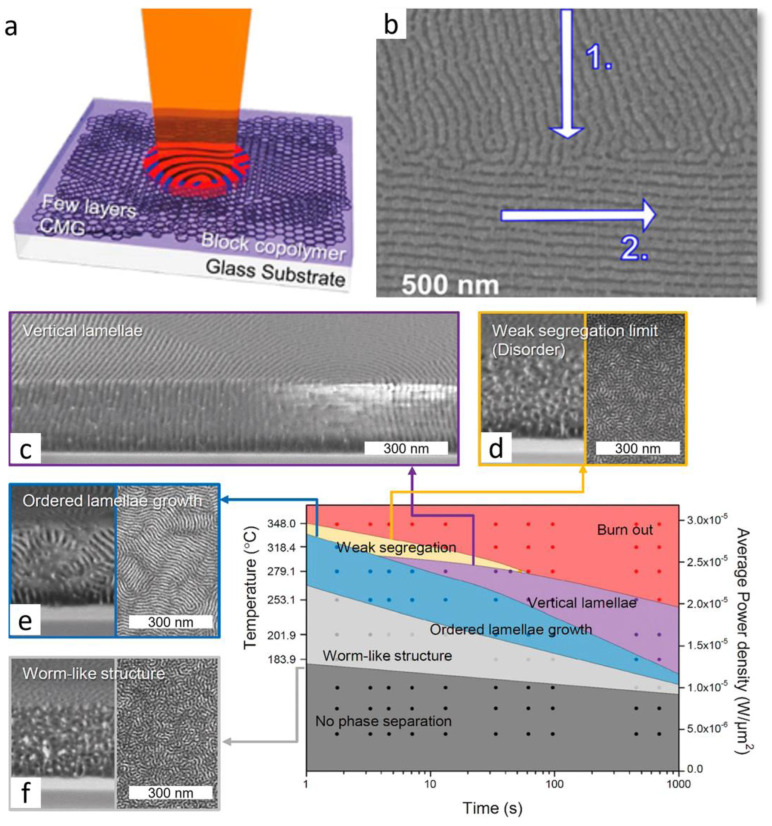
(**a**) Schematic of the laser zone annealing process. (**b**) Local patterning via soft-shear laser zone annealing (SS-LZA) of PS-*b*-PMMA, note the perpendicular junction from the two orthogonal laser sweeps [91]. (**c**–**f**) BCP self-assembly of the PS-*b*-PMMA blended with the short neutral random copolymer chains of PS-*r*-PMMA (7:3 weight ratio) under the transient laser photothermal treatment at increasing intervals of heat and time [98]. Adapted with permission from references [91,98].

As such, Majewski et al. achieved the long range order via ‘soft -shear’ laser zone annealing (SS-LZA) with a high-power (3 W) green (532 nm) laser focused to a sharp line using a mirror in a vacuum chamber [91]. By placing a sheet of PDMS on top of the PS-*b*-PMMA film, the large thermal expansion coefficient of PDMS induces a predictable tangent stress array along the laser sweep, as shown in the schematic of Figure 6a [91]. (The thermal expansion coefficients (×10^−4^ K^−1^) of PS, PMMA, and PDMS at 180 °C, are 5.84, 5.7, and 9.53, respectively [97]). Figure 6b displays the local patterning of PS-*b*-PMMA with the SS-LZA effect of the induced stress via SEM, noting the perpendicular intersection patterning from two orthogonal laser sweeps, with the use of the beam-masking edge (170 nm processed with a single sweep at 320 μm/s [laser line FWHM = 93 μm]) [91].

Similarly, CO_2_ pulse lasers are often employed for this process, yet at a higher laser power, such that many BCPs were significantly damaged after multiple scans and caused warping within the film [95,96,99]. At very slow velocities of LZA, degradation also occurred, demonstrating the influence of kinetics on the morphology [91,96]. Hence the use of higher temperatures on larger time scales for more rapid reordering is limited by the thermal degradation of the BCP make-up (polystyrene degrades at 330 °C, and poly(methyl methacrylate) [91,95]. However, reports suggest that high temperatures up to 1000 °C can be employed at millisecond time frames, and the reordering of the BPC assembly in PS-*b*-PMMA can effectively remove the defects in a linearly aligned lamellae film pattern, with little to no detectable degradation of the BCP [91,95,96].

The phase diagram outlined in Figure 6c–f displays the BCP metastability behavior in the thick films on the chemically modified graphene (CMG) (transmittance = 89.2%), displaying the laser power density average vs. the irradiation time [98]. Controlling these parameters creates a series of BCP morphologies that deviate from its equilibrium lamellae structure. The BCP morphology in Figure 6f displays a random worm-like kinetically trapped metastable phase. These worm-like structures in the BCP have been attributed to low laser power densities of less than 1.0 × 10^−5^ W/μm^2^, independent of the irradiation time, where the available thermal energy is insufficient for the defect annihilation [98]. When the laser power density is increased, a higher-ordered structure fingerprint-like region of the lamellae growth is produced (Figure 6e) [98]. Further increasing the laser power densities, leading to the vertical lamellae (Figure 6d), briefly before the weak segregation morphologies can be detected (Figure 6c) [98]. Beyond 2.7 × 10^−5^ W/μm^2^, the BCP begins to transition from the ordered to disordered before the film experiences burnout (thermal degradation) at around 2.92 × 10^−5^ W/μm^2^ [91,98]. The relationship between the laser power density and the irradiation time demonstrates the tunability of the morphology via LZA on the nanodomain alignment by using a localized heat source method [91,96,98,100].

#### 3.2.2. Cold Zone Annealing

**Figure 7:**
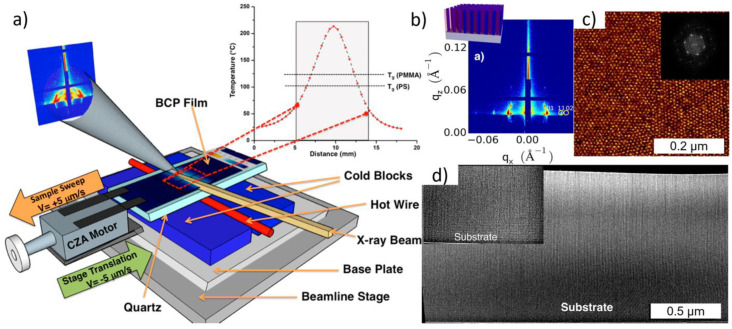
(**a**) Schematic of a cold zone annealing sharp (CZA-S) assembly for in-situ GISAXS experiments. (**b**) GISAXS image analogous with a hexagonal lattice with oriented cylinder axes out of the plane. (**c**) Hexagonally packed cylindrical morphology of PS-b-PMMA after CZA-S [102]. (**d**) TEM image of a cross-sectional view of the film with a bimodal orientation Inset: Parallel cylinder orientation near the substrate base [102]. Adapted with permission from reference [102].

Cold zone annealing (CZA) originates from the metallurgy field and semiconductor industry with the goal to restrict the grain growth in a narrow melt process that heavily relies on temperature gradients to refine the material [101]. This process is shown in Figure 7a where a BCP film moves along the cold-hot-cold CZA assembly, using a step motor, while a beamline stage translates in the opposite direction [102]. Saumil et al. utilized this setup to probe the phase transition of the BCP films in situ [102]. From this, a temperature gradient curve can be assembled, which in turn determines the BCP order-disorder transition temperatures [94,101,102]. Additionally, temperature gradient curves predetermine whether the zone annealing is CZA or hot zone annealing and allows for the targeting of different morphologies along this curve. In the case of the cylinder forming poly(styrene-block-methyl methacrylate) (PS-*b-*PMMA) films, when the temperature gradient field strength is broad, it demonstrates the long rang order with horizontally orientated cylinders [101]. At sharp gradients (CZA-S), the vertically orientated hexagonally close-packed cylinders are preferred [101]. Figure 7c displays an AFM image of the highly ordered hexagonal packing of the vertical orientated cylinders from a CZA-S processed PS-*b-*PMMA (35-*b*-12 kg mol^−1^) film [102].

Moreover, at shorter time intervals during the CZA-S process, i.e., the high annealing velocities, the BCP thin film may avoid the chain stretching and/or compression by conforming to the vertical orientation, independent of thickness [101,102]. At high velocities, it has been suggested that PMMA and PS are kinetically forbidden to wet the surface and air, respectively, thus relieving the chain stretching by adopting the vertical orientation [101]. Additionally when more time was allowed for the CZA process, PMMA wets the surface and PS wets the air forming the horizontal ordered terrace structures [101]. This was attributed to the relationship between the lamellae grain coarsening from the cooling and the simultaneously increasing interaction parameter [102]. However, future investigations of these interactions may be needed to gain a better understand of the rapid kinetic interactions. Nonetheless, the metastable orientational control of the BCP thin films obtained via CZA-S annealing may provide a high-throughput avenue for functional templates in the future [93,101].

The relevant measure of the order corresponds to the time the sample spent at a temperature greater than the T_g_ of both blocks (126 °C T_g_, PMMA). GISAXS has been shown to have distinct thermal signals from the as-cast to post-processed films in the annealing process [102]. Based on the scattering patterns and the incidence angle, the GISAXS for the as-cast generally displays a very weak scattering pattern, suggesting a low ordered film. As the BCP aligns, the peaks shift, become sharper, and give rise to consistent lattice spacing that suggests the ordering of the microphase separated PMMA microdomains [102]. Figure 7b shows GISAXS pattern corresponding to the sharp peak spacing association to the hexagonal lattice with a consistent spacing of 23.8 nm [94,102].

A cross-sectional TEM image in Figure 7d demonstrates the bimodal orientation of the cylinders near the substrate. Although most cylinders are vertical, visible perpendicular horizontal cylinders are observed near the surface of the substrate throughout the 1 µm thick films [102]. It has been suggested that the sharp thermal gradient affects the thermal expansion within the BPC [103]. The resulting vertical cylinders over the horizontal cylinders are assumed to originate from the vertical strain induced by the sharp thermal gradients [102,103]. The similar controlled reordering of the PS-*b-*PMMA BCP phases have been observed in previous reports using CZA-S annealing techniques, that confirm the process and success of these film alignments [93,94,101,104]. By tuning the CZA-S process, this study was able to induce ca. 95% hexagonally close packed vertical cylinders in the sub-100 nm PS-*b-*PMMA (35-*b*-12 kg mol^−1^) BCP film thickness region [102]. This mixed morphology of perpendicular cylinders near the quartz substrate has been previously observed, yet not fully understood [102,103]. It has been postulated that the PS-*b-*PMMA films on silicon substrates, heated during the CZA-S process, mobilizes the chains allowing PMMA to migrate towards SiO2, forming a similar pattern [103]. It has also been suggested that the PMMA phase continually attempts to wet the surface while energized [93,101].

### 3.3. Limitations and Future Prospects

The key advantage of microwave-assisted annealing is the ability to form these well-ordered patterns in periods of less than a minute, and hence be consistent with large scale manufacturing. It can also be used as a promising method to explore the metastable phases of different BCPs. Understanding the kinetics of the metastable morphologies can uncover new avenues for BCP thin film applications. However, this method does not come without limitations. One study proposes that there is a time lag for microwave irradiated thin films to reach the desired temperature [77]. This proposes that organization may take place during this time and the kinetics of self-assembly may completely differ from the anticipated. Furthermore, microwave annealed films have not been subjected to rapid quenching, again leaving the morphologies to further rearrange. This aspect should be carefully considered when applying microwave-annealing as a potential method.

Comparably, LZA and cold zone annealing (CZA) often both use an adapted transient laser heating system as a heat source [102]. Additionally, the same method is generally applied, using a consistent heat source and a constant velocity to direct the self-assembly within the BCP films. In fact, both adaptations heavily rely on the same parameters, such as power density, velocity, and shape of the laser–this dictates morphology of the BCP. Yet these laser heating methods are still subject to limitations, such as controlling the morphology, thermal annealing time for self-assembling, warping, and thermal degradation. Studies have displayed that a higher power density with millisecond annealing times can give rise to the reordering of the BCP at temperatures above the thermal degradation limits. Using the CZA methods, a sharper temperature gradient peak over which the sample is heated and cooled, essentially limits the laser-heated highly energized portion of the BCP to a time-dependent rapid cooldown, similar to rapid quenching [102]. This, in-turn, may also help eliminate the possible warping and burnout of the BCP from the high temperature during the rapid reordering by kinetically trapping the BCP in a metastable phase.

The utilization of LZA, CZA and microwave-assisted annealing for soft materials is starting to become more well-established, especially for the potential application in the microelectronics and lithography domains [88,100,102]. Though laser heating methods are becoming more accurate at directing the long-range order within the BCP thin films, the chance of localized defects from high temperatures is greater than in microwave-assisted annealing [84,91]. The fabrication of selective vertical or horizontal orientations of the BCP cylinders is constantly being modified [102]. As such, there have been many endeavors in the pursuit of accomplishing the directed self-assembly process in BCPs in post-preparation heating processes [102].

## 4. Conclusions

In this review, we discussed how different annealing methods can be utilized to obtain a wide range of metastable phases during the BCP self-assembly. Furthermore, we highlighted the significance of overcoming the kinetic barriers and rapid quenching on preserving the metastable morphologies and different methods used in that regard. Solvent-induced methods are excellent for high χ systems where kinetic barriers tend to be overwhelming for thermal annealing. The presence of solvent molecules alters the effective volume fraction of each block, depending on the affinity, providing a direct method for screening the BCP phase space. Solvent annealing, however, typically requires an elaborate apparatus that could face challenges in large scale processing. Rapid thermal annealing methods are more promising in this regard, even allowing for local phase transformation in the case of laser induced heating. The kinetics of quenching the target morphologies is also faster, compared to solvent systems, when considering the phonon vs. diffusion-controlled transport. Nevertheless, these methods are restricted to the metastability in the orientation, likely due to the lack of volume control, hence, limiting the accessible phases. Overall, there is great promise in exploring the kinetics and mechanisms of the novel processing techniques for controlling the self-assembly and orientational transformation in the metastable states.

## Data Availability

No new data were created or analyzed in this study. Data sharing is not applicable to this article.
